# Parvovirus B19‐associated myocarditis in children: A systematic review of clinical features, management and outcomes

**DOI:** 10.1111/eci.70102

**Published:** 2025-07-28

**Authors:** Giacomo Veronese, Giada Colombo, Andrea Garascia, Rachele Adorisio, Ezio Bonanomi, Enrico Ammirati

**Affiliations:** ^1^ Pediatric Intensive Care Unit ASST Papa Giovanni XXIII Bergamo Italy; ^2^ Department of Cardiothoracic Surgery Heart and Vascular Centre, Maastricht University Medical Centre Maastricht The Netherlands; ^3^ De Gasperis Cardio Center, Niguarda Hospital Milan Italy; ^4^ Heart Failure, Transplant and Mechanical Assist Device Unit IRCCS Bambino Gesù Children's Hospital Rome Italy; ^5^ University of Milan‐Bicocca, School of Medicine and Surgery Monza Italy

**Keywords:** cardiogenic shock, paediatric myocarditis, parvovirus B19, viral myocarditis

## Abstract

**Background:**

Parvovirus B19 (PVB19) has emerged as a relevant etiologic agent of paediatric myocarditis, particularly during recent epidemiological surges in Europe and the United States. Despite increasing recognition, current knowledge remains fragmented, and standardised diagnostic and therapeutic strategies are lacking.

**Methods:**

We conducted a systematic review of the literature up to May 2025, including 40 studies encompassing 53 individual case reports, 107 patients from registry‐based cohorts, and 4 tissue‐based investigations.

**Results:**

Clinical presentation was frequently fulminant, with cardiogenic shock, severe left ventricular dysfunction, and need for mechanical circulatory support in up to 47% of cases. Mortality rates ranged from 10% to 30%, with heart transplantation rates varying between 5% and 42% across cohorts. Diagnosis relied primarily on blood polymerase chain reaction (PCR), while serology showed limited diagnostic utility. Histological confirmation via endomyocardial biopsy (EMB) was variably applied across studies, and myocardial viral load quantification was reported in only one study. Case series and cohort studies confirmed early age of onset (median 16–24 months), respiratory or gastrointestinal prodromes, and poor outcomes in fulminant presentations. Tissue‐based studies revealed high myocardial PVB19 loads in acute myocarditis, particularly in infants, but also demonstrated viral persistence in asymptomatic individuals, complicating causal inference. Immunomodulatory therapy was administered in up to 58% of cases, although its clinical impact remains uncertain due to heterogeneity in treatment protocols. No antiviral treatments have been evaluated to date.

**Conclusion:**

These findings highlight the need for standardised diagnostic criteria incorporating PCR, serology, imaging, and, where appropriate, EMB and viral load assessment. Given recent epidemiologic surges and high morbidity, prospective multicentre studies and surveillance efforts are urgently required to refine clinical algorithms and improve outcomes in paediatric PVB19 myocarditis.

## Graphical abstract

This systematic review summarizes clinical features, diagnostic strategies and outcomes of paediatric myocarditis associated with Parvovirus B19. Affected children are typically under 3 years old and often present with fulminant symptoms, severe cardiac dysfunction and a high need for ICU‐level support or mechanical circulatory assistance. In‐hospital mortality reaches up to 30%, and heart transplantation is not uncommon. Current evidence is limited by diagnostic heterogeneity and a lack of virus‐specific therapies, highlighting the need for standardized approaches and outbreak surveillance strategies.

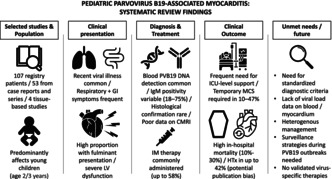

## 1 INTRODUCTION

Paediatric acute myocarditis has an estimated annual incidence of approximately 2 per 100,000 children.^1^ While the aetiology is diverse, viral infections remain the predominant cause, with a progressive epidemiological shift over recent decades from adenovirus and enterovirus toward Parvovirus B19 (PVB19) and human herpesvirus 6.^2^, ^3^ During the COVID‐19 pandemic, a distinctive cardiogenic shock phenotype emerged in children with multisystem inflammatory syndrome, further underscoring the evolving clinical landscape of paediatric myocarditis.^4^ Mortality varies significantly by aetiology, ranging from 0% in adolescents with COVID‐19‐related myocarditis to nearly 31% in neonates with enteroviral forms.^5^, ^6^, ^7^

PVB19 is a small, non‐enveloped, single‐stranded DNA virus and the only known human pathogen within the Parvoviridae family.^8^, ^9^, ^10^ It is highly transmissible via respiratory droplets and poses a particular risk to individuals in close contact with children. The clinical spectrum of PVB19 infection ranges from mild viral illnesses, including erythema infectiosum (fifth disease), to severe complications such as transient aplastic crisis and hydrops fetalis.^11^, ^12^

PVB19 has also been increasingly implicated as a potential cause of acute myocarditis.^13^ Although its pathogenic mechanisms remain incompletely understood, immune‐mediated injury is presumed to play a major role. In children, PVB19‐associated myocarditis, though relatively uncommon, may manifest as fulminant acute myocarditis, characterised by the rapid onset of severe heart failure and cardiogenic shock, requiring advanced pharmacological or mechanical circulatory support (MCS).^14^

Recent reports from the United States and Europe have documented a marked resurgence in PVB19 circulation,^15^, ^16^, ^17^ prompting health advisories from both the U.S. Centers for Disease Control and Prevention (CDC)^18^ and the European Centre for Disease Prevention and Control (ECDC).^19^ Notably, CDC surveillance data indicate a sharp rise in PVB19 IgM seroprevalence, from <3% in 2022–2023 to 10% in 2024, peaking at 40% among children aged 5–9 years.^20^ Parallel epidemiological trends have been observed in France and Italy,^15^, ^21^, ^22^ with a rising number of severe paediatric myocarditis cases attributed to PVB19 reported since January 2024.^23^, ^24^

Despite increasing recognition, consolidated evidence on paediatric PVB19‐associated myocarditis remains limited. Diagnostic strategies are heterogeneous, and no standardised treatment protocols exist. This systematic review aims to synthesise existing clinical, virological and histological evidence from case reports, series, cohort studies and tissue‐based investigations to provide a comprehensive characterisation of paediatric myocarditis associated with PVB19. By integrating available data, this review seeks to inform clinical decision‐making and highlight priorities for future research.

## 2 MATERIALS AND METHODS

### 2.1 Literature search

A systematic literature search was conducted in accordance with the Preferred Reporting Items for Systematic Reviews and Meta‐Analyses (PRISMA) guidelines. Searches were performed in PubMed, Embase, and Web of Science from database inception to May 2025, with no restrictions on language or publication year. The search strategy included terms related to Parvovirus B19 (e.g. ‘Parvovirus B19’, ‘B19 virus’, ‘human parvovirus’, ‘erythrovirus’, ‘fifth disease’), myocarditis (e.g. ‘myocarditis’, ‘cardiac inflammation’, ‘myocardial inflammation’, ‘inflammatory cardiomyopathy’, ‘viral myocarditis’) and paediatric populations (e.g. ‘child’, ‘adolescent’, ‘infant’, ‘paediatric’, ‘neonate’, ‘under 18’). Both controlled vocabulary (e.g. MeSH in PubMed, Emtree in Embase) and free‐text keywords were used, tailored to the syntax and indexing of each database. Boolean operators (AND, OR) were applied to combine search terms, and strategies were iteratively refined to maximize sensitivity and specificity. Example search strings included: PubMed (‘Parvovirus B19’ [MeSH Terms] OR ‘Parvovirus B19’ [Title/Abstract] OR ‘B19 virus’ [Title/Abstract] OR ‘human parvovirus’ [Title/Abstract] OR ‘erythrovirus’ [Title/Abstract] OR ‘fifth disease’ [Title/Abstract] OR ‘fifth disease virus’ [Title/Abstract]) AND (‘Myocarditis’ [MeSH Terms] OR ‘Myocarditis’ [Title/Abstract] OR ‘cardiac inflammation’ [Title/Abstract] OR ‘myocardial inflammation’ [Title/Abstract] OR ‘inflammatory cardiomyopathy’ [Title/Abstract] OR ‘viral myocarditis’ [Title/Abstract] OR ‘heart inflammation’ [Title/Abstract] OR ‘cardiomyopathy’ [Title/Abstract]) AND (‘Child’ [MeSH Terms] OR ‘Adolescent’ [MeSH Terms] OR ‘Infant’ [MeSH Terms] OR ‘Paediatrics’ [MeSH Terms] OR ‘paediatric’ [Title/Abstract] OR ‘children’ [Title/Abstract] OR ‘infant’ [Title/Abstract] OR ‘neonate’ [Title/Abstract] OR ‘newborn’ [Title/Abstract] OR ‘young people’ [Title/Abstract] OR ‘under 18’ [Title/Abstract] OR ‘school‐age’ [Title/Abstract]). Search strings for Embase and Web of Science followed equivalent logic. Detailed search strings for each database are provided in Appendix S1. References were exported to EndNote (Clarivate Analytics), and duplicates were removed prior to screening.

Eligible studies reported on patients under 18 years of age with a diagnosis of myocarditis based on clinical, imaging, or histological criteria consistent with the American College of Cardiology or Dallas definitions^25^, ^26^ and confirmed PVB19 infection via polymerase chain reaction (PCR) and/or serologic testing (IgM or IgG). We included case reports, case series, retrospective cohorts, registry‐based studies and tissue‐based investigations. Two reviewers (G.V. and G.C.) independently screened all titles, abstracts and full‐text articles, with discrepancies resolved by consensus. Reference lists of selected articles were reviewed for additional relevant studies. Reasons for exclusion included studies not focusing on acute myocarditis associated with PVB19, studies enrolling only adult patients, animal models, laboratory‐based experiments or those investigating molecular mechanisms of PVB19 without associated clinical data. Additional exclusion criteria were letters to the editor, commentaries, narrative reviews, non‐English language publications and studies with unavailable full text or insufficient clinical detail.

**FIGURE 1:**
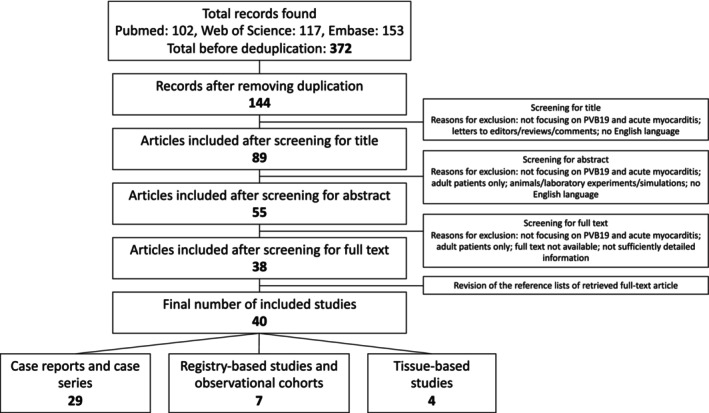
PRISMA flow diagram of the systematic review process for paediatric Parvovirus B19‐associated myocarditis. The diagram illustrates the identification, screening, eligibility, and inclusion phases of study selection. A total of 372 records were identified across PubMed, Embase, and Web of Science. After removal of duplicates (228 records), 144 records underwent title and abstract screening. Following full‐text review, 40 studies were included in the systematic review: 29 case reports/series, 7 registry‐based or observational cohorts, and 4 tissue‐based studies. Reasons for exclusion at each stage are provided in the diagram. PVB19, Parvovirus B19.

Of the 144 articles initially identified through database searching, 40 met the inclusion criteria and were included in the final analysis (Figure 1). These comprised 29 case reports or small case series, 7 registry‐based or observational cohort studies, and 4 tissue‐based studies (via endomyocardial biopsy [EMB] or autopsy), some of which lacked detailed patient‐level information.

For each included study, we extracted data on demographics, medical history, clinical presentation, electrocardiographic and imaging findings (echocardiography, cardiac magnetic resonance [CMRI]), laboratory and virologic parameters (PCR, serology), treatment strategies, including supportive and immunomodulatory therapies and outcomes such as recovery, heart transplant (HTx), need for MCS or death.

### 2.2 Statistical Analysis

A dedicated database was constructed to collect clinical information from paediatric patients with PVB19‐associated myocarditis identified in case reports and series. Only studies reporting sufficient individual‐level data were included in this analysis. For each case, variables were extracted using a standardized form and included age, sex, clinical features, diagnostic modality (PCR, serology), left ventricular ejection fraction (LVEF), need for inotropic support, mechanical ventilation, MCS, immunomodulatory therapy and clinical outcome. Data were obtained from article texts, tables, and figures. Descriptive statistics were used to summarise cohort characteristics. For registry‐based studies and retrospective cohorts without access to individual patient data, aggregate outcomes were collected and described narratively. Given the substantial heterogeneity in study design, populations and outcome reporting, a formal meta‐analytic pooling of data and assessment of heterogeneity were not performed. Special attention was given to study methodology, diagnostic consistency and the virologic criteria used. We performed a study quality appraisal using the Joanna Briggs Institute Critical Appraisal Checklists for Case Reports^27^ for studies reporting sufficient individual‐level data, grading reporting quality as high, moderate or low based on criteria met (Appendix S1). While this provided a structured assessment of reporting completeness, formal risk of bias scoring and quantitative synthesis were not feasible due to the descriptive nature of the studies. Tissue‐based studies were analysed separately due to their distinct design and limited clinical granularity.

All data analysed in this review are derived from previously published studies and are available in the public domain.

## 3 RESULTS

### 3.1 Observational evidence from paediatric cohorts

**FIGURE 2:**
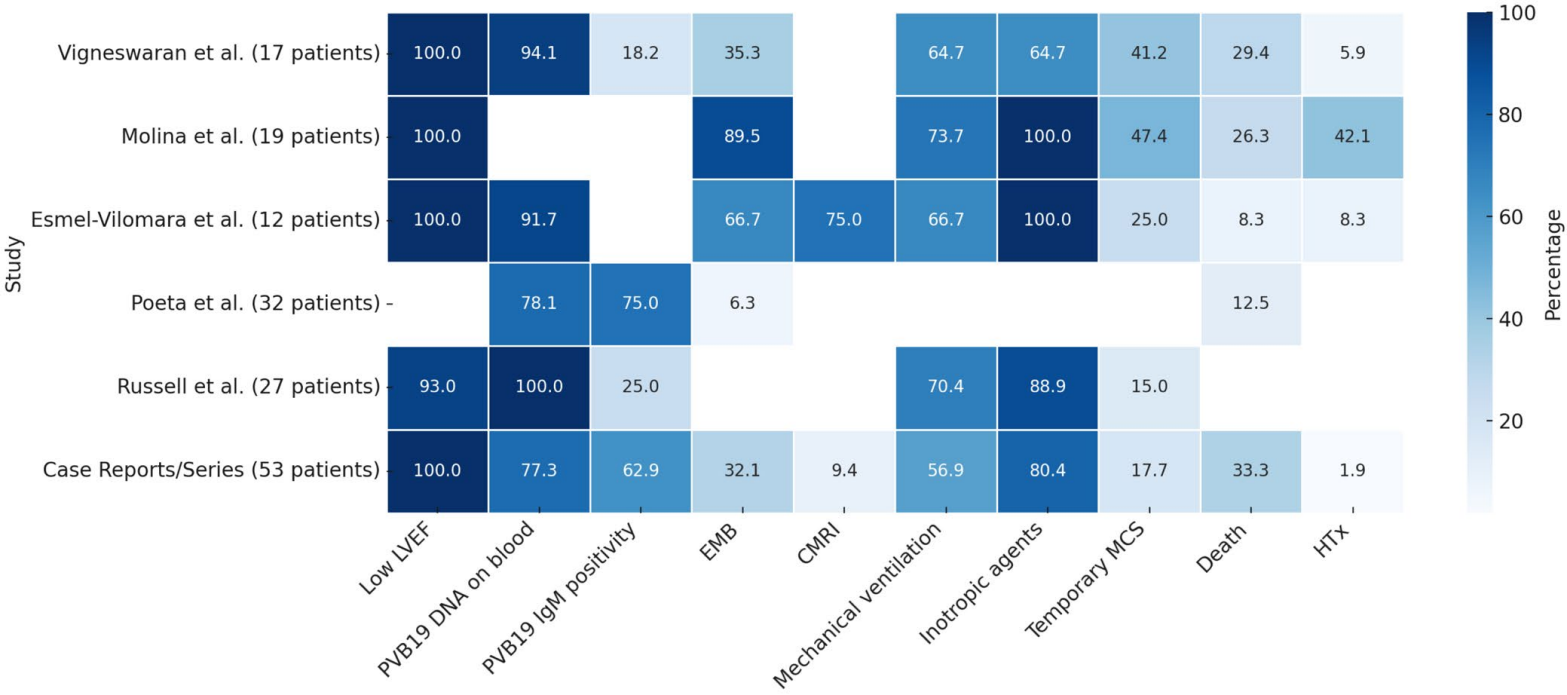
Summary heatmap of key clinical, diagnostic, management, and outcome features in paediatric patients with Parvovirus B19‐associated myocarditis across selected studies. The heatmap depicts the proportion (expressed as percentage) of patients presenting with each feature within individual studies. Features included low left ventricular ejection fraction (LVEF), detection of PVB19 DNA in blood, IgM positivity, use of endomyocardial biopsy (EMB), cardiac magnetic resonance imaging (CMRI), mechanical ventilation, inotropic support, temporary mechanical circulatory support (MCS), death, and heart transplantation (HTx). Studies represented comprise five retrospective cohort or registry‐based investigations and a pooled cohort of 53 paediatric patients derived from published case reports and case series. Data highlight variability in diagnostic approaches, management strategies, and outcomes across settings. Colour intensity corresponds to the reported percentage, as indicated by the accompanying scale bar. CMRI, cardiac magnetic resonance imaging; EMB, endomyocardial biopsy; HTx, heart transplantation; IgM, immunoglobulin M; LVEF, left ventricular ejection fraction; MCS, mechanical circulatory support; PVB19, Parvovirus B19.

Seven retrospective or registry‐based studies reported cohorts of paediatric patients with PVB19‐associated myocarditis.^14^, ^23^, ^24^, ^28^, ^29^, ^30^, ^31^ Three of these studies originated from the same research group^29^, ^30^, ^31^ and analysed overlapping cohorts, each focusing on different diagnostic or therapeutic aspects. To avoid redundancy, only the original cohort publication was included in the main comparative analysis.^29^ A summary of clinical features and outcomes across the cohorts is reported in Table 1 and Figure 2.

**TABLE 1:** Comparative overview of paediatric cohorts with Parvovirus B19‐associated myocarditis.

Author	Type of study	Patients	Age and sex	t‐MCS	PVB19 diagnosis	Histology available	IM therapy	Outcomes	Strengths	Limitations
Vigneswaran et al.	Single‐center retrospective (2003–2009) Endemic setting	17 Mainly clinical diagnosis	Range: 0.4 y‐15.4 y (median 1.3 y) 52.9% female	7 ECMO (41.2%)	Blood PCR 16/17 (94.1%) Tissue PCR performed in 7/17; 6/7 positive (85.7%) 2/11 were PVB19 IgM positive	41.1% (7/17 patients) 6 EMB 1 post‐mortem Histological evidence of lymphocytic infiltration was seen in 5/6 EMB	9 IVIG (52.9%)	5 deaths (29.4%) 1 HTx (5.8%)	Detailed patient‐level data, diagnosis primarily clinical with PCR on blood	Single‐centre, small sample size, no viral load, histological data were partially available, virologic diagnosis was based primarily on blood PCR (qualitative)
Molina et al.	Multicenter retrospective (2 centers) (2005–2008) Endemic setting	19 Mainly histological diagnosis	Age range: 6 m−15 y (median 16 m) 74% F	9 MCS (47.3%)	PCR on myocardial tissue (89.6%) Tracheal aspirate (5.2%) and pleural fluid (5.2%) PCR PCR on blood not reported	17/19 (89.5%) All lymphocytic myocarditis	Not reported	5 deaths (26.3%) 8 HTx (42.1%)	Detailed data, highest EMB rate, with diagnosis confirmed histopathologically, multicenter design	Small sample size, no viral load, absence of blood‐based virological testing, selection bias (severe forms undergoing EMB)
Esmel‐Vilomara et al.	Single‐center retrospective (2007–2021) Endemic setting	12 PVB19‐myocarditis among 53 myocarditis cases (22.6%) Mainly histological/CMRI diagnosis	Age range: 7 m–3 y (median 21.5 m) 41.6% F	3 MCS (25%)	PCR on blood 11/12 (91.7%) PCR on myocardial tissue 9/12 (75%)	9/12 (75%) 8 EMB 1 post‐mortem All lymphocytic myocarditis	Steroids + IFN‐β 7/12 (58.3%)	1 death (8.3%) 1 HTx (8.3%)	Diagnosis confirmed either histopathologically or by CMRI; detailed histological data; single center prevalence among a cohort of paediatric myocarditis	Single‐centre, small sample size, qualitative PCR; heterogeneous diagnostic workup
Poeta et al.	Multicenter retrospective (12 centers) 1/01/2024–31/10/2024 Outbreak setting	32 PVB19‐myocarditis among a cohort of 65 paediatric myocarditis (49.2%) Mainly clinical diagnosis	Median 6.1 y (IQR 2.2 y – 13.7 y) 39/65 M	6/65 VA ECMO Breakdown by PVB19 status not specified for MCS	PCR on blood 25/32 (78.1%) Positive IgM serology 24/32 (75%) PCR on myocardial tissue 2/32 (6.2%)	3 EMB/65 No details on histology	Overall 28/65 (43%) 7/65 steroids 8/65 IVIG, 13/65 steroids + IVIG 9/65 anakinra breakdown by PVB19 status not specified for therapies	4 deaths (12.5%)	Multicenter study; prevalence among a cohort of paediatric myocarditis during a PVB19 outbreak	Diagnosis of myocarditis was primarily clinical, limited histological confirmation, incomplete virological testing, breakdown by PVB19 status not specified for therapies/MCS
Russell et al.	Multicenter retrospective (2019–2024) Outbreak setting	27 8 cases (2019–2023) + 19 cases (2024) Mainly clinical diagnosis	Median 21 m (IQR 4 m‐14 y) 55% female	4 ECMO (15%)	Blood PCR 100% Viral load in blood ranged from 17,700 to 708,000 IU/mL 5 (25%) IgM positive	Not reported 1 had positive PCR result for PVB19 on EMB	3 IVIG (11.1%)	93% ICU admission/89% inotropic support/70% IMV 2024: 14/19 discharged, 5/19 still hospitalized 2019–2023: 88% discharged	Virologically confirmed cases with quantitative PCR on blood, recent data on a national PVB19 outbreak	Small sample size, histological data not available, short/incomplete follow‐up

Abbreviations: CMRI, cardiac magnetic resonance imaging; EMB, endomyocardial biopsy; F/M, female/male; HTx, heart transplantation; IFN‐β, interferon beta; IM, immunomodulatory; IMV, invasive mechanical ventilation; IQR, interquartile range; IVIG, intravenous immunoglobulin; PCR, polymerase chain reaction; PVB19, Parvovirus B19; t‐MCS, temporary mechanical circulatory support; VA ECMO, veno‐arterial extracorporeal membrane oxygenation; y/m, years/months.

#### 3.1.1 Evidence during periods without documented increased PVB19 circulation (endemic setting)

Vigneswaran et al.^28^ reported 17 cases identified between 2003 and 2009 at a single UK centre. PVB19 DNA was detected in the blood of 16/17 patients and one (1/17) post‐mortem myocardial biopsy. In those tested, 2/11 were PVB19 IgM positive and 8/8 were PVB19 IgG positive. Six patients underwent EMB and histological evidence of lymphocytic infiltration was seen in 5/6 patients. The median age was 1.3 years (range 0.4–15.4) and 52.9% were female. All patients presented with severe heart failure (mean fractional shortening 15 ± 3%), and 41.2% required extracorporeal membrane oxygenation (ECMO). Intravenous immunoglobulin (IVIG) therapy was administered in 52.9% of patients. One patient underwent HTx (5.8%), and five died (29.4%). Notably, all deaths occurred among patients with fulminant presentation, ST‐segment elevation on ECG, or prodromal symptoms of less than 48 h, underscoring the poor prognosis of fulminant cases.

In a dual‐center US study, Molina et al.^14^ retrospectively analysed 19 children diagnosed with PCR‐verified PVB19 myocarditis between 2005 and 2008. The median age was 16 months, and 74% were female. All patients presented with severe LV dysfunction (median LVEF 24%), and MCS was required in 47.3%. Respiratory symptoms were reported in 89% of patients, and gastrointestinal manifestations such as emesis or diarrhoea occurred in 63%. Eight children underwent HTx (42.1%), and five died (26.3%). Only six patients (31.6%) survived without HTx, and full recovery of systolic function was achieved in just five. ST‐segment elevation and high LV end‐diastolic dimension z‐scores were associated with adverse outcomes. For 17 patients (89.5%), the diagnosis was confirmed predominantly with myocardial tissue sampling. The remaining two patients did not undergo EMB, and PVB19 PCR was detected by tracheal aspirate and pleural fluid, respectively. All biopsies showed lymphocytic myocarditis. No data on immunosuppressive therapy were provided.

Esmel‐Vilomara et al.^29^ reported a single‐centre prospective cohort of 12 PVB19‐confirmed myocarditis episodes over 14 years (2007–2021). The median age was 21.5 months (range 7–36 months), and 41.6% were female. Cardiogenic shock was the initial presentation in 58.3% of cases. Severe LV dysfunction was universal, and 25% required MCS. Immunomodulatory therapy with corticosteroids and interferon beta was used in 58.3% of cases, all of whom showed significant improvement; six of seven achieved complete recovery. Overall HTx‐free survival was 83.3%, higher than previous series, possibly due to early intervention and immunotherapy. Myocarditis was diagnosed by histology in 9 patients (75%) and by CMRI in 8/9 patients (88.9%). PVB19 was confirmed by PCR on blood or myocardial tissue.

#### 3.1.2 Evidence during periods of increased PVB19 circulation (outbreak setting)

Two recent multicenter studies have highlighted distinct outbreaks of paediatric PVB19‐associated myocarditis across Europe, underscoring the emerging role of PVB19 as a prominent etiologic agent during epidemic periods. A multicenter Italian surveillance study by Poeta et al.^23^ described 65 paediatric cases of acute myocarditis during a 10‐month outbreak in 2024, of which 32 (49.2%) had confirmed PVB19 infection. The median age of PVB19‐positive cases was 6.1 years (IQR 2.2–13.7), and 60% were male. In the entire cohort, 9.2% of patients required ECMO. Among those with confirmed PVB19 infection, the in‐hospital mortality rate was 12.3%. Most PVB19‐positive children had a preceding viral‐like illness, with respiratory symptoms reported in 56%, gastrointestinal symptoms in 31% and fever in 41%. Diagnosis of myocarditis was primarily clinical, with CMRI or histologic confirmation available only in selected cases. Virologic confirmation among the 32 PVB19‐positive patients was based on blood PCR in 25 cases (78.1%), IgM serology in 24 cases (75.0%) and myocardial tissue PCR in only 2 cases (6.2%). Only 3 EMBs were performed across the cohort (2 PVB19‐positive), and no histologic details were reported. Immunomodulatory therapy was used in 43% of patients overall, including corticosteroids, IVIG and anakinra, although subgroup‐specific data were not disclosed. Importantly, granular patient‐level data were only available for a subset of cases, and virologic testing was incomplete in 17% of the total population. Complementing these findings, Russell et al.^24^ reported on a UK multicenter cohort of 27 children diagnosed with PVB19 myocarditis between 2019 and 2024, with a surge observed in early 2024. The median age was 21 months, and severe left ventricular systolic dysfunction was common (median LVEF 25%), often accompanied by respiratory symptoms and haemodynamic compromise. All patients were diagnosed via blood PCR, while IgM seropositivity was observed in only 25% of those tested. Quantitative viral load data were available in a subset of patients, ranging from 17,700 to 708,000 IU/mL, although no direct correlation with disease severity was reported. ICU admission was required in 93% of cases, 15% required ECMO and three experienced cardiac arrest. Notably, immunomodulatory therapy was used in only 3 cases. Overall, 7 of 8 (88%) of those admitted before 2024 survived to discharge; 14 of those admitted in 2024 were discharged with ongoing follow‐up; and the remaining 5 were still inpatients at the time of writing.

Taken together, these studies confirm that PVB19 myocarditis can present with fulminant clinical features and carries substantial risk of mortality and need for advanced MCS. However, heterogeneity in diagnostic criteria, therapeutic approaches, and outcome reporting, as well as small cohort sizes, limits broader conclusions. Most studies lacked standardised virologic quantification (e.g., PVB19 DNA copy number) or consistent histological confirmation, limiting inter‐study comparability and external validity. Additional variability stems from incomplete reporting of supportive therapies, unclear long‐term outcomes and inconsistent definitions of myocarditis diagnosis (clinical vs. histologic vs. imaging‐based).

### 3.2 Evidence from case reports and case series

A total of 15 case series^32^, ^33^, ^34^, ^35^, ^36^, ^37^, ^38^, ^39^, ^40^, ^41^, ^42^, ^43^, ^44^, ^45^, ^46^ (average number of patients 2, overall number 39 patients) and 14 case reports^47^, ^48^, ^49^, ^50^, ^51^, ^52^, ^53^, ^54^, ^55^, ^56^, ^57^, ^58^, ^59^, ^60^ for a total of 53 collected cases were included in the current review. All studies were published between 1998 and May 2025 (Table 2 and Table S1). Overall study quality is reported in Table S2. The median age at presentation was 24 months (IQR, 11–60), with 79.3% of patients under 7 years of age. Males represented 45.3% of the population. None had a prior history of myocarditis, and only two cases reported a familial history of cardiomyopathy.

**TABLE 2:** Clinical presentation, diagnostic approach, treatment strategies, and outcomes in paediatric Parvovirus B19‐associated myocarditis: Summary of case reports and case series.

Overall cases, *n*	53
Demographics, available data, *n* (%)	53 (100)
Age, months, median (Q1–Q3)	24 (11–60)
Age <7 years, *n* (%)	42 (79.3)
Male, *n* (%)	24 (45.3)
Main clinical manifestations, available data, *n* (%)	49 (92.5)
Days between onset and admission, median (Q1–Q3)	1 (1–2)
Worsening dyspnea, *n* (%)	20 (40.8)
Cardiac arrest/cardiogenic shock, *n* (%)	11 (22.5)
Chest pain/palpitation, *n* (%)	1 (2.1)
Nausea/vomiting/diarrhoea/loss of appetite, *n* (%)	10 (20.4)
Asthenia/lethargy, *n* (%)	4 (8.2)
Fever on admission, *n* (%)	11 (22.5)
Prodromal symptoms, available data, *n* (%)	49 (92.5)
Time between onset and admission, days, median, (Q1–Q3)	5 (1–8)
Gastrointestinal symptoms, *n* (%)	16 (32.7)
Respiratory symptoms, *n* (%)	33 (67.4)
Fever before admission, *n* (%)	18 (36.7)
Viral exanthem before admission, *n* (%)	16 (32.7)
Known or suspected V disease before admission, *n* (%)	14 (28.6)
ECG on admission, available data, *n* (%)	18 (34.0)
Described abnormal ST‐T segment, *n* (%)	13 (72.2)
Life threatening arrhythmias before/on admission, available data, *n* (%)	51 (96.2)
Cardiac arrest requiring CPR, *n* (%)	5 (9.8)
Troponin T/I, available data, *n* (%)	17 (32.1)
Increased troponin T/I, *n* (%)	16 (94.1)
Evidence of PVB19 infection, available data, *n* (%)	53 (100)
Serology performed, *n* (%)	35 (66.0)
Serology, IgG positivity, *n* (%)	27 (77.1)
Serology, IgM positivity, *n* (%)	22 (62.9)
PCR DNA on plasma performed, *n* (%)	41 (77.4)
PCR DNA on plasma identified, *n* (%)	41 (100.0)
Echocardiography on admission, available data, *n* (%)	26 (49.1)
LVEF, %, median (Q1–Q3)	19 (15–26)
Definite diagnosis of myocarditis based on, available data, *n* (%)	53 (100)
Clinical criteria, *n* (%)	21 (39.6)
CMRI criteria, *n* (%)	5 (9.4)
Histologic criteria, *n* (%)	27 (50.9)
Histology available, overall *n* (%)	29 (54.7)
Postmortem examination, *n* (%)	12 (22.6)
EMB, *n* (%)	17 (32.1)
Lymphocytic myocarditis, *n* (%)	25 (86.2)
PVB19 identified on myocardial tissue, *n* (%)	5 (17.2)
Treatment, available data, *n* (%)	51 (96.2)
Type of hemodynamic support	
None, *n* (%)	10 (19.6)
Inotrope use only, *n* (%)	32 (62.8)
Any temporary MCS, *n* (%)	9 (17.7)
VA ECLS, *n* (%)	8 (15.7)
IABP, *n* (%)	1 (2.0)
Inotrope use overall, *n* (%)	41 (80.4)
Invasive mechanical ventilation, *n* (%)	29 (56.9)
Any immunomodulating therapy, *n* (%)	26 (50.9)
IVIG, *n* (%)	20 (76.9)
Steroids, *n* (%)	14 (53.9)
Anakinra, *n* (%)	1 (3.8)
Other, *n* (%)	8 (30.8)
Outcome, available data, *n* (%)	51 (96.2)
Death, *n* (%)	17 (33.3)
HTx, *n* (%)	1 (1.9)

Abbreviations: CMRI, cardiac magnetic resonance imaging; CPR, cardiopulmonary resuscitation; ECG, electrocardiogram; EMB, endomyocardial biopsy; HTx, heart transplant; IABP, intra‐aortic balloon pump; Ig, immunoglobulins; IVIG, intravenous immunoglobulin; LVAD, left ventricular assist device; LVEF, left ventricular ejection fraction; MCS, mechanical circulatory support; PCR, polymerase chain reaction; PVB19, Parvovirus B19; PVB19, parvovirus B19; VA ECLS, veno‐arterial extracorporeal life support.

Prodromal symptoms typically began approximately 5 days before hospitalization (IQR, 1–8) and were most often respiratory (67.4%) or gastrointestinal (32.7%) in nature. Fever was present in 36.7% of patients prior to admission and persisted in 22.5% at presentation. A viral exanthem was noted in 32.7%; fifth disease was confirmed or suspected in fourteen cases (28.6%).

Leading symptoms developed a median of 1 day before hospital admission (IQR, 1–2). Dyspnea was the most common presenting feature (40.8%), while critical presentations such as cardiac arrest or overt cardiogenic shock were reported in 22.5%. Gastrointestinal symptoms (nausea, vomiting, diarrhoea or poor oral intake) were documented in 20.4% of patients.

Electrocardiographic abnormalities were reported in all 18 patients for whom ECG data were available, with ST‐segment elevations frequently observed (72.7%). Cardiac arrest requiring cardiopulmonary resuscitation occurred in 5 cases (9.8%). Cardiac biomarkers, when reported, were markedly elevated.

PVB19 DNA was detected by PCR on plasma in 100% of tested cases (41/41). IgG and IgM seropositivity were reported in 77.1% and 62.9% of tested patients (overall number, 35), respectively, highlighting the limited diagnostic value of acute serologies.

Echocardiographic data revealed depressed LV function in all patients for whom ejection fraction was reported, with a median LVEF of 19% (IQR, 15–26). Diagnosis was established through CMRI in 5 patients (9.4%, all meeting Lake Louise Criteria), by histopathological analysis in 27 patients (50.9%) and based on clinical criteria in the remaining cases. EMB was performed in 17 patients (32.1%), while post‐mortem histologic confirmation was obtained in 12 (22.6%). Myocardial detection of PVB19 DNA was confirmed in only 5 cases.

Supportive care was frequently required. Inotropic therapy was used in 80.4% of patients, mechanical ventilation was required in 56.9%, and a temporary MCS was employed in 17.7% of patients.

Immunomodulatory therapy was administered in 50.9% of cases, with IVIG being the most common intervention (76.9% of treated patients), followed by corticosteroids, used in 53.9% of treated patients.

The overall survival rate free from HTx or durable ventricular assist device (LVAD) implantation was 66.7%. Seventeen patients (33.3%) died, one underwent HTx (1.9%) and none required LVAD.

### 3.3 Viral genome detection and histopathologic evidence in broader paediatric cohorts

In addition to case reports and cohort studies with detailed clinical data, several retrospective studies have investigated the association between PVB19 and paediatric myocarditis using molecular, histological and post‐mortem tissue analyses. While these studies did not provide granular clinical‐level information, they offer relevant insights into viral prevalence, pathogenesis and tissue distribution patterns. A synthesis of tissue‐based investigations, including studies utilising endomyocardial biopsy or post‐mortem myocardial analysis, is presented in Table 3.

**TABLE 3:** Comparative summary of tissue‐based paediatric studies on myocardial Parvovirus B19 detection.

Author	Type of study	Patients	Age	PVB19 diagnosis	Strengths	Limitations
Pelz et al.	Single center retrospective (2004–2023)	1366 histologically confirmed acute (306) or chronic (1060) lymphocytic myocarditis	<16 y, many <2 y	PVB19 DNA was detected in: 131/306 (43%) of children with acute lymphocytic myocarditis. 149/1060 (14%) with chronic lymphocytic myocarditis	Large paediatric cohort with systematic EMB analysis Integrated molecular (quantitative PCR, ISH) and histological evaluation	Clinical correlates limited, no detailed data on treatment, outcomes or MCS use
Gagliardi et al.	Single center retrospective (2001–2013)	63 paediatric patients with acute HF undergoing EMB and qualitative PCR testing for cardiotropic viruses	Median age: 2.7 y; range: 0.1–17.5 y	23 patients (36.5%) were diagnosed with myocarditis—14 with acute lymphocytic myocarditis and 9 with borderline myocarditis PVB19 was detected in myocardial tissue 10/23 cases (43.4%)	Systematic use of EMB in all HF patients Viral PCR testing performed uniformly on all EMB samples	No detailed clinical data on PVB19‐positive patients No PVB19 viral load or quantitative PCR reported
Nielsen et al.	Retrospective forensic autopsy‐based study (1992–2010)	112 myocarditis or borderline myocarditis at autopsy 84 control cases with morphologically normal hearts, 33/112 B19 myocarditis, 37/84 B19 infections	3 w – 77 y (12 cases <12 y) in myocarditis group Control group (1 case <12 y)	PVB19 DNA in myocardial tissue was detected by PCR in 29% (33/112) of myocarditis cases and in 44% (37/84) of controls Serological data (available for 57 myocarditis cases): only 1 case had IgM+ (suggesting acute infection), while 63% were IgG+ and 33% were IgG−	Large autopsy cohort with histologically confirmed myocarditis Addition of serological testing	Paediatric data limited No viral load quantification Post‐mortem analysis
Schowengerdt et al.	Single center retrospective (1989–1997)	360 suspected myocarditis 200 transplant rejections 250 control group	Myocarditis: age range from 1 day to 21 y (mean 7.4 y) Transplant group: aged 6 months to 21 y (mean 8.1 y)	PVB19 detected in myocardial tissue by PCR Suspected myocarditis: 3/360 (0.8%) Paediatric transplant recipients: 6/200 (3%) Control group: 0/250 (0%)	Large paediatric cohort with systematic EMB sampling Inclusion of control and transplant groups for comparison Histological diagnosis (Dallas criteria) used for myocarditis confirmation	Very low number of PVB19‐positive cases No data on viral load or quantitative PCR No detailed patient data

Abbreviations: EMB, endomyocardial biopsy; HF, heart failure; IgM/IgG, immunoglobulin M/immunoglobulin G; ISH, in situ hybridisation; MCS, mechanical circulatory support; PCR, polymerase chain reaction; PVB19, Parvovirus B1; w/m/y, weeks/months/years.

Schowengerdt et al.^61^ conducted a historical retrospective cohort study of 360 paediatric patients (mean age 7.4 years) evaluated for suspected myocarditis between 1989 and 1997. All patients underwent EMB, and a total of 391 myocardial tissue samples (including initial and follow‐up biopsies) were analysed for PVB19 DNA using qualitative PCR. Three patients (0.8%) had myocardial PVB19 positivity, all of whom met Dallas criteria for lymphocytic myocarditis. Among these three, one patient died, one developed dilated cardiomyopathy and one recovered, suggesting potential variability in clinical trajectories. Notably, PVB19 was not detected in any of the 250 control biopsies from patients with cardiomyopathy of known aetiology, supporting a possible pathogenic role.

Pelzl et al.^62^ conducted a large single‐centre retrospective study of 1366 paediatric patients (<16 years) with histologically confirmed acute or chronic lymphocytic myocarditis based on EMB between 2004 and 2023. PVB19 DNA was detected in 131 of 306 children with acute myocarditis (43%) and in 149 of 1060 with chronic myocarditis (14%), making it the most frequently identified cardiotropic virus in the cohort. Virologic analyses included nested and quantitative PCR on myocardial tissue, with mean viral loads significantly higher in acute compared to chronic myocarditis (15,395 vs. 2386 copies/μg DNA). In situ hybridisation confirmed active replication of PVB19 within endothelial cells of arterioles and venules. Infants and toddlers (≤2 years) displayed particularly high viral loads and dense T‐cell infiltration in the myocardium, suggesting an age‐dependent inflammatory response. The authors described two fatal cases: one infant with fulminant myocarditis and another adolescent with sudden cardiac death, both with high myocardial PVB19 loads and histologic confirmation of lymphocytic myocarditis. Despite the robust pathological and virological characterisation, the study did not report clinical outcomes such as MCS, immunotherapy or HTx need. Nonetheless, it provides compelling evidence for a direct pathogenic role of PVB19 in paediatric myocarditis, especially in younger children.

Gagliardi et al.^63^ conducted a retrospective cohort study of 63 paediatric patients admitted with acute heart failure between 2001 and 2013, all of whom underwent EMB with PCR testing for cardiotropic viruses. According to the Dallas criteria, 23 patients (36.5%) were diagnosed with myocarditis, 14 with acute lymphocytic myocarditis and 9 with borderline myocarditis, while the remaining 40 were diagnosed with dilated cardiomyopathy. Among patients with biopsy‐confirmed myocarditis (including both acute and borderline forms), PVB19 was detected in 10 cases (43.4%). Although this study provides valuable data on the distribution of PVB19 across histological subtypes, it does not offer detailed clinical information regarding the presentation, treatment or outcomes of PVB19‐positive patients. Furthermore, no data were reported on PVB19 viral load or quantitative PCR results, limiting the interpretability of the virologic findings.

Nielsen et al.^64^ conducted a large retrospective autopsy study in Denmark to assess the prevalence of viral genomes in myocardial tissue, comparing 112 individuals diagnosed with myocarditis at post‐mortem with 84 control cases who died of non‐cardiac causes and had structurally normal hearts. PVB19 DNA was detected in 29% of myocarditis cases and 44% of controls using PCR on formalin‐fixed, paraffin‐embedded tissue. Among the 57 myocarditis cases with available serologic data, only one showed IgM positivity, indicating recent infection, whereas 63% were IgG positive and 33% IgG negative. These findings suggest that PVB19 DNA may persist in myocardial tissue regardless of histological inflammation or acute infection and support the interpretation of PVB19 as a potential bystander in many post‐mortem cases. The lack of correlation between PCR positivity and serologic evidence of acute infection highlights the limitations of relying solely on PCR for etiologic diagnosis in myocarditis, particularly in autopsy settings. These findings underline the possibility of PVB19 persistence in the myocardium, complicating causal attribution.

These investigations underscore the frequent detection of PVB19 in paediatric myocardium, particularly among infants and young children. However, findings range from clear pathogenic roles (with high viral loads and inflammation) to incidental presence, complicating causal inference. The absence of standardized viral load thresholds, variable histologic correlation, and lack of uniform clinical data highlight the need for integrated, prospective, multicentre studies to refine diagnostic and aetiological criteria.

## 4 DISCUSSION

This systematic review demonstrates that PVB19‐associated acute myocarditis in children disproportionately affects infants and toddlers and frequently follows a fulminant clinical course. Across case reports and series, the median age was 24 months, and even lower in registry‐based studies (e.g., 15 months in Vigneswaran et al.^28^ and 21 months in Esmel‐Vilomara et al.^29^ and Russel et al.^24^), highlighting a possible age‐related vulnerability. This may reflect immature immune responses, higher endothelial viral tropism or increased exposure in early childhood.

The clinical picture is often critical (Figure 2). In individual‐level data, median LVEF at admission was approximately 19%, and 80.4% of patients required inotropic support. Mechanical ventilation was needed in over half of the cases, and the need for MCS was consistently reported. Survival free from HTx or durable MCS was 64.8% in case reports and series, whereas in‐hospital mortality in registry‐based cohorts ranged from 10% to 30%, substantially higher than that typically observed in paediatric myocarditis, highlighting the considerable burden of adverse outcomes.

These findings align with the 2022 review by Keramari et al.,^65^ which described a similarly severe phenotype among 32 PVB19 myocarditis cases. Symptoms included tachycardia (69%), ST‐segment elevation (31.5%) and arrhythmias (15.6%), with many requiring intensive care unit‐level support. Our findings reinforce that PVB19 myocarditis often follows a fulminant trajectory, particularly in younger children. In this context, a recently identified ECG pattern, characterised by peaked P waves, low QRS voltages and abnormal repolarisation, has demonstrated high specificity for PVB19‐associated myocarditis, potentially aiding early recognition and differentiation from other aetiologies.^31^

Compared to non‐PVB19 paediatric myocarditis,^26^, ^66^, ^67^, ^68^ PVB19 positive cases appear markedly more severe. Reported mortality has reached 30% in PVB19‐associated myocarditis, in contrast to 1.6% in a large Finnish myocarditis registry.^69^ Other studies report mortality rates of 16% and HTx‐free survival near 87%.^70^, ^71^, ^72^ Age appears to be a central prognostic factor, with MYKKE data^73^ and our review indicating worse outcomes in infants compared to older children. These differences likely reflect a combination of diagnostic delays, distinct host responses and lower myocardial reserve in early life.

Clinically, PVB19‐associated myocarditis is often insidious in onset and diagnosis remains challenging. The absence of the classic erythema infectiosum rash was notable, present in approximately 30% of cases. Instead, nonspecific prodromes such as respiratory, gastrointestinal or febrile symptoms were common, potentially delaying suspicion and diagnosis. ECG abnormalities, elevated troponin and NT‐proBNP levels, and reduced LVEF were frequently observed. CMRI was valuable for supporting diagnosis when performed. Although infrequently performed, EMB confirmed lymphocytic myocarditis in several cases and remains the diagnostic gold standard; however, its routine application is limited in paediatric settings, particularly outside tertiary care centres.

Virologic confirmation of PVB19 relies primarily on PCR‐based DNA detection on either blood or myocardial tissue, but its interpretation remains challenging. Positivity may reflect either acute infection or persistent viremia, even in the absence of IgM seroconversion. Serologic testing was frequently inconclusive, and histologic correlation with PCR results was inconsistent across studies. Pelzl et al.^62^ demonstrated that high myocardial viral loads, particularly in young children, are associated with acute lymphocytic myocarditis, supporting a pathogenic role. In contrast, autopsy studies such as Nielsen et al.^64^ highlighted that PVB19 DNA may persist in myocardial tissue without active inflammation, suggesting a possible bystander phenomenon and complicating aetiologic attribution, especially in the absence of viral load quantification or histopathologic confirmation.

At present, there is no virus‐specific therapy for PVB19‐associated myocarditis. Immunomodulatory treatment, primarily IVIG and corticosteroids, was used in nearly half of the cases and may offer benefit in fulminant forms, although robust evidence is lacking. Agents such as interferon beta or anakinra were reported in isolated cases only. No study systematically evaluated treatment response or viral load changes, limiting inference. The heterogeneity in treatment approaches underscores the need for clinical trials or at least prospective observational registries. A recent cohort by Gran et al.^30^ suggested that combined treatment with interferon‐beta and corticosteroids may improve outcomes in paediatric patients with PVB19‐associated myocarditis, supporting the potential role of this therapeutic strategy in selected cases.

The recent surge in paediatric PVB19‐associated myocarditis, particularly in 2024, may reflect post‐pandemic shifts in population‐level immunity, resulting in increased susceptibility among young children.^74^ The parallel rise in IgM seroprevalence and reports from Italy and France support this epidemiological signal.^15^, ^22^ These developments highlight the need for enhanced virologic surveillance, unified diagnostic criteria and multidisciplinary awareness, especially among paediatricians, cardiologists, intensivists and virologists.

## 5 LIMITATIONS

This review has several important limitations. First, it is based almost entirely on retrospective and observational data, which carry inherent risks of selection bias, variable data quality and publication bias. Second, there was considerable heterogeneity in diagnostic criteria, particularly regarding myocarditis definitions (clinical vs. histological vs. imaging‐based), and inconsistency in virologic testing, limiting comparability across studies. While one study assessed myocardial viral load^62^ and only a few reported quantitative PVB19 DNA levels in plasma,^24^ most lacked blood‐based viral profiling, highlighting a gap in distinguishing active infection from latent persistence. Third, information on treatment protocols, response and long‐term outcomes was frequently missing, preventing definitive conclusions. Given the substantial heterogeneity in study design, populations and outcome reporting, this review is primarily descriptive and narrative in nature; a formal meta‐analytic pooling of data and assessment of heterogeneity were not performed. Finally, despite including the largest body of PVB19 paediatric myocarditis data to date, our findings remain hypothesis‐generating and require validation in prospective studies.

## 6 FUTURE PERSPECTIVES

**FIGURE 3:**
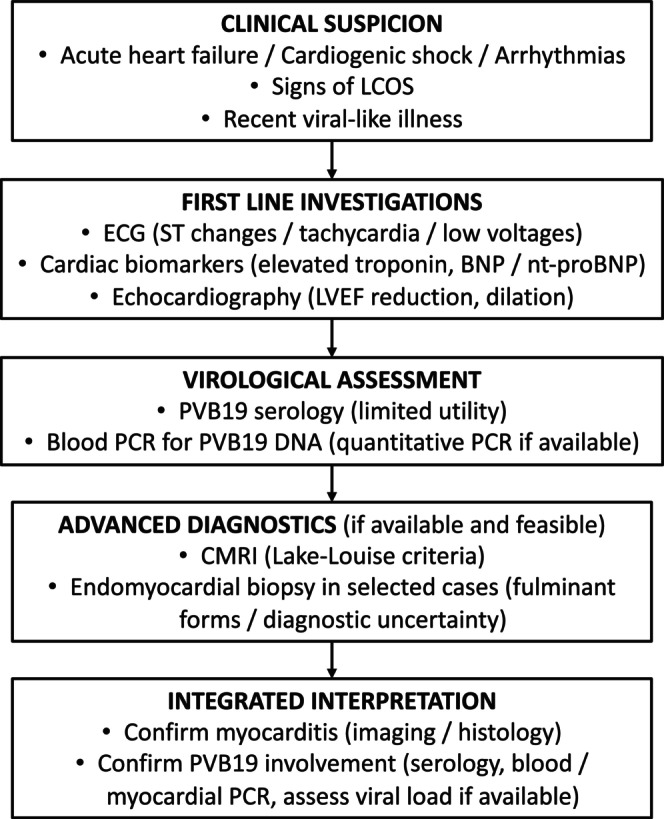
Simplified diagnostic algorithm for paediatric Parvovirus B19‐associated myocarditis. The figure outlines a stepwise approach for the evaluation of suspected cases, starting from clinical suspicion (acute heart failure, cardiogenic shock, arrhythmias or low cardiac output signs following a viral‐like illness), first‐line investigations (ECG, cardiac biomarkers, echocardiography) and virological assessment (serology and blood PCR for PVB19 DNA, preferably quantitative). Integrated interpretation combines clinical, imaging, and virological data to confirm myocarditis and PVB19 involvement. Advanced diagnostics such as endomyocardial biopsy are reserved for selected cases, particularly fulminant forms or where diagnostic uncertainty persists. BNP, brain natriuretic peptide; CMRI, cardiac magnetic resonance imaging; LCOS, low cardiac output signs; LVEF, left ventricular ejection fraction; nt‐proBNP, N‐terminal pro‐B‐type natriuretic peptide; PCR, polymerase chain reaction; PVB19, Parvovirus B19.

Several key areas warrant focused attention to advance the understanding and management of PVB19‐associated myocarditis in children. First, prospective multicenter registries with standardized data collection are urgently needed to capture the full clinical spectrum, clarify age‐specific risk profiles, and better quantify incidence and outcomes. Second, integration of viral load quantification, tissue tropism analysis and host immunogenetic profiling may help differentiate pathogenic infection from incidental viremia, especially in cases where histological evidence is lacking. The development of composite diagnostic algorithms combining PCR, serology, imaging and biomarkers could enhance early identification and risk stratification. A simplified diagnostic algorithm summarising key steps for clinical and virologic evaluation is provided in Figure 3, aiming to support future standardisation efforts.

From a treatment standpoint, the field would benefit from randomized or at least well‐designed observational studies evaluating the efficacy of immunomodulatory agents, including IVIG, corticosteroids, interferon beta and targeted cytokine inhibitors such as anakinra. The potential role of antiviral therapy remains unexplored and may become relevant if viral replication proves to be a primary driver of myocardial injury in a subset of patients.

Finally, considering recent epidemiologic trends, surveillance systems integrating virologic, serologic and clinical data at the population level could play a critical role in early outbreak detection and response. As the burden of PVB19 myocarditis becomes more apparent, these forward‐looking strategies will be essential for reducing diagnostic delays, optimising management and ultimately improving outcomes in this high‐risk paediatric population.

## 7 CONCLUSION

PVB19‐associated myocarditis is an increasingly recognized but underdiagnosed cause of severe cardiac dysfunction in young children, particularly in infants and toddlers. This review, representing the most comprehensive aggregation of paediatric PVB19 myocarditis cases to date, highlights its early age of onset, nonspecific clinical features, diagnostic challenges and high morbidity and mortality. Delayed recognition is common due to the absence of classical signs (e.g. fifth disease rash), the limited sensitivity of serologic testing, and the heterogeneous application of diagnostic tools such as CMRI and EMB. While PCR on blood is widely used, it cannot reliably distinguish between active infection and persistent viremia, particularly when viral load quantification or histopathologic confirmation is lacking. As no targeted antiviral therapies are currently available, management relies on immunomodulation (IVIG, corticosteroids) and advanced supportive measures, including MCS, which are frequently required. Outcomes remain poor in a significant proportion of cases, particularly in younger children and those with fulminant presentation.

The recent epidemiologic surge of PVB19 in several countries, coinciding with a rise in severe myocarditis cases, calls for urgent action. Prospective research efforts, multicenter registries and standardised diagnostic protocols are essential to better characterise the disease, refine therapeutic approaches and improve prognosis. As the clinical relevance of PVB19 myocarditis continues to emerge, coordinated efforts across paediatric cardiology, virology and intensive care are needed to reduce diagnostic delays, inform treatment decisions and optimise outcomes in this vulnerable population.

## AUTHOR CONTRIBUTIONS

G.V. and E.A. conceptualised the study and coordinated the review process. G.V. and G.C. conducted the literature search and data extraction. G.V. and G.C. assessed study eligibility and performed qualitative data synthesis. G.V. and G.C. drafted the manuscript. A.G., E.A., E.B. and R.A. critically revised the manuscript for important intellectual content. All authors reviewed and approved the final version of the manuscript.

## FUNDINGS

Dr. Enrico Ammirati reports receiving funding from the Italian Ministry of Health (GR‐2019‐12368506) as principal investigator of the investigator‐driven MYTHS (Myocarditis Therapy with Steroids) trial, and from the Italian Ministry of Health and NextGenerationEU (PNRR‐MAD‐2022‐12376225). No specific funding was received for the present study.

## CONFLICT OF INTEREST STATEMENT

Dr. Enrico Ammirati reports serving as a consultant for Lexeo, AstraZeneca and Cytokinetics. The other authors declare no conflicts of interest.

## Supporting information


Data S1.

## Data Availability

All data analysed in this review are derived from previously published studies and are available in the public domain.
