# Supplementary material for: Parvovirus B19‐associated myocarditis in children: A systematic review of clinical features, management and outcomes
Source: Eur J Clin Invest. 2025 Jul 28;55(11):e70102. doi: 10.1111/eci.70102 (PMC12517251; doi:10.1111/eci.70102)
Supplement: Supplementary file 1 — Data S1. [file ECI-55-e70102-s001.docx]

**Parvovirus B19-Associated Myocarditis in Children: A Systematic Review of Clinical Features, Management, and Outcomes**

G Veronese and G Colombo et al.

**Appendix S1**

**Search Strategies**

**PubMed**

("Parvovirus B19"[MeSH Terms] OR "Parvovirus B19"[Title/Abstract] OR "B19 virus"[Title/Abstract] OR "human parvovirus"[Title/Abstract] OR "erythrovirus"[Title/Abstract] OR "fifth disease"[Title/Abstract] OR "fifth disease virus"[Title/Abstract]) AND ("Myocarditis"[MeSH Terms] OR "Myocarditis"[Title/Abstract] OR "cardiac inflammation"[Title/Abstract] OR "myocardial inflammation"[Title/Abstract] OR "inflammatory cardiomyopathy"[Title/Abstract] OR "viral myocarditis"[Title/Abstract] OR "heart inflammation"[Title/Abstract] OR "cardiomyopathy"[Title/Abstract]) AND ("Child"[MeSH Terms] OR "Adolescent"[MeSH Terms] OR "Infant"[MeSH Terms] OR "Pediatrics"[MeSH Terms] OR "pediatric"[Title/Abstract] OR "children"[Title/Abstract] OR "infant"[Title/Abstract] OR "neonate"[Title/Abstract] OR "newborn"[Title/Abstract] OR "young people"[Title/Abstract] OR "under 18"[Title/Abstract] OR "school-age"[Title/Abstract])

**Embase**

('parvovirus b19'/exp OR 'parvovirus b19':ti,ab OR 'b19 virus':ti,ab OR 'human parvovirus':ti,ab OR 'erythrovirus':ti,ab OR 'fifth disease':ti,ab OR 'fifth disease virus':ti,ab) AND ('myocarditis'/exp OR 'myocarditis':ti,ab OR 'cardiac inflammation':ti,ab OR 'myocardial inflammation':ti,ab OR 'inflammatory cardiomyopathy':ti,ab OR 'viral myocarditis':ti,ab OR 'heart inflammation':ti,ab OR 'cardiomyopathy':ti,ab) AND ('child'/exp OR 'adolescent'/exp OR 'infant'/exp OR 'pediatrics'/exp OR 'pediatric':ti,ab OR 'children':ti,ab OR 'infant':ti,ab OR 'neonate':ti,ab OR 'newborn':ti,ab OR 'young people':ti,ab OR 'under 18':ti,ab OR 'school-age':ti,ab)

**Web of Science**

TS=("Parvovirus B19" OR "B19 virus" OR "human parvovirus" OR "erythrovirus" OR "fifth disease" OR "fifth disease virus") AND TS=("myocarditis" OR "cardiac inflammation" OR "myocardial inflammation" OR "inflammatory cardiomyopathy" OR "viral myocarditis" OR "heart inflammation" OR "cardiomyopathy") AND TS=("pediatric" OR "children" OR "infant" OR "neonate" OR "newborn" OR "young people" OR "under 18" OR "school-age")

**Supplementary table 1.** Summary of individual case reports of pediatric Parvovirus B19-associated myocarditis: clinical features, management, and outcomes.

|  |  | **Age, mo** | **Sex** | **Diagnosis** | **HTx** | **t-MCS** | **PCR DNA on blood** | **CMR** | **EMB** | **IM**  **therapy** | **Last FU** |
| --- | --- | --- | --- | --- | --- | --- | --- | --- | --- | --- | --- |
| 1 | Izquierdo-Blasco J et al. | 24 | M | H | N | Y | Y | NR | N | NR | D |
| 2 | Canales Siguero D et al. | 6 | M | CMR | N | N | Y | Y | Y | Y | A |
| 3 | Canales Siguero D et al. | 9 | M | CMR | N | N | Y | Y | N | Y | A |
| 4 | Jain P et al. | 24 | M | CL | N | N | Y | N | N | N | A |
| 5 | Jain P et al. | 4 | M | CL | N | N | Y | N | N | N | A |
| 6 | Adda J et al. | 180 | F | CMR | N | N | Y | Y | N | N | A |
| 7 | Dina J et al. | 18 | F | H | N | N | Y | N | N | N | D |
| 8 | Dina J et al. | 9 | M | CL | N | N | Y | N | N | N | A |
| 9 | Dina J et al. | 30 | F | CL | N | N | Y | N | N | N | A |
| 10 | Veronese G et al. | 12 | F | H | N | N | Y | N | Y | Y | A |
| 11 | Hu HY et al. | 84 | M | H | N | N | Y | N | N | N | D |
| 12 | Hu HY et al. | 11 | F | H | N | N | Y | N | N | N | D |
| 13 | Koehl B et al. | 36 | M | H | N | N | Y | N | N | N | D |
| 14 | Koehl B et al. | 60 | M | H | N | N | Y | N | N | N | D |
| 15 | Papadogiannakis N et al. | 11 | F | H | N | N | Y | N | N | N | D |
| 16 | Nigro G et al. | 7 | F | CL | N | N | Y | N | Y | Y | A |
| 17 | Nigro G et al. | 12 | F | H | N | N | NR | N | Y | Y | A |
| 18 | Nigro G et al. | 18 | F | H | N | N | Y | N | Y | Y | A |
| 19 | Butin M et al. | 72 | M | CL | N | N | Y | N | Y | Y | A |
| 20 | Munro K et al. | 24 | M | H | N | N | Y | N | Y | Y | A |
| 21 | Munro K et al. | 84 | F | H | N | Y | Y | N | Y | Y | A |
| 22 | Munro K et al. | 19 | M | H | N | N | Y | N | Y | Y | D |
| 23 | Enders G et al. | 156 | M | H | Y | N | Y | N | Y | Y | A |
| 24 | Enders G et al. | 84 | F | H | N | N | Y | N | Y | N | A |
| 25 | Murry CE et al. | 60 | F | H | N | N | Y | N | N | N | D |
| 26 | Dina J et al. | 60 | F | H | N | N | Y | N | N | N | D |
| 27 | Krishnamurti L et al. | 39 | F | CL | N | Y | Y | N | N | Y | A |
| 28 | Rohayem et al. | 132 | M | H | N | N | Y | N | N | N | D |
| 29 | Zack F et al. | 60 | F | H | N | N | Y | N | N | N | D |
| 30 | Beghetti M et al. | 48 | F | H | N | N | Y | N | N | N | D |
| 31 | Beghetti M et al. | 108 | M | CL | N | N | NR | N | N | Y | A |
| 32 | McMahon CJ et al. | 48 | M | CL | N | N | Y | N | N | Y | A |
| 33 | McMahon CJ et al. | 36 | M | CL | N | N | Y | N | N | Y | A |
| 34 | O’Connell MJ et al. | 7 | M | CL | N | N | NR | N | N | N | A |
| 35 | Floyd A et al. | 168 | M | CMR | N | N | Y | Y | N | N | A |
| 36 | Ajmi H et al. | 36 | F | CL | N | N | NR | N | N | N | A |
| 37 | Spartalis M et al. | 204 | F | CMR | N | Y | Y | Y | N | Y | A |
| 38 | Saleh M et al. | 12 | M | CL | N | N | Y | N | N | Y | A |
| 39 | Saleh M et al. | 9 | F | CL | N | N | Y | N | N | Y | A |
| 40 | Amabile N et al. | 21 | NR | CL | N | N | Y | N | N | Y | A |
| 41 | Amabile N et al. | 12 | NR | CL | N | N | Y | N | N | Y | A |
| 42 | Simpson KE et al. | <12 | NR | CL | NR | NR | Y | N | NR | NR | NR |
| 43 | Simpson KE et al. | 13 | NR | CL | NR | NR | Y | N | NR | NR | NR |
| 44 | Ferro V et al. | 144 | F | H | N | Y | Y | N | Y | N | D |
| 45 | Ferro V et al. | 7 | F | H | N | Y | Y | N | Y | Y | A |
| 46 | Ferro V et al. | 8 | M | H | N | Y | Y | N | Y | Y | A |
| 47 | Ferro V et al. | 108 | M | CL | N | N | Y | N | N | Y | A |
| 48 | Ferro V et al. | 12 | F | H | N | N | Y | N | Y | Y | A |
| 49 | Ferro V et al. | 24 | M | H | N | Y | Y | N | Y | Y | A |
| 50 | Ferro V et al. | 36 | M | CL | N | N | Y | N | N | N | A |
| 51 | Wahjudi T et al. | 7 | F | CL | N | Y | Y | N | N | NR | D |
| 52 | Wahjudi T et al. | 9 | F | H | N | N | NR | N | Y | NR | D |
| 53 | Wahjudi T et al. | 10 | F | H | N | N | Y | N | Y | NR | D |

**Abbreviations:** mo, months; HTx, heart transplant; t-MCS, temporary-mechanical circulatory support; PCR, polymerase chain reaction; CMR, cardiac magnetic resonance; EMB; endomyocardial biopsy; IM, immunomodulatory; FU, follow-up; M, male; F, female; H, histology; CL, clinical; Y, yes; N, no; NR, not reported; D, death; A, alive.

**Supplementary table 2.** Quality appraisal of individual pediatric Parvovirus B19-associated myocarditis case reports using the JBI Critical Appraisal Checklist.

|  |  | **ITEM 1**  Were patient’s demographic characteristics clearly described? | **ITEM 2**  Was the patient’s history clearly described and presented as a timeline? | **ITEM 3**  Was the current clinical condition of the patient on presentation clearly described? | **ITEM 4**  Were diagnostic tests or assessment methods and the results clearly described? | **ITEM 5**  Was the intervention(s) or treatment procedure(s) clearly described? | **ITEM 6**  Was the post-intervention clinical condition clearly described? | **ITEM 7**  Were adverse events (harms) or unanticipated events identified and described? | **ITEM 8**  Does the case report provide takeaway lessons? | **Overall study quality (according to JBI** **Critical Appraisal Checklists) *** |
| --- | --- | --- | --- | --- | --- | --- | --- | --- | --- | --- |
| 1 | Izquierdo-Blasco J et al. | Y | Y | Y | Y | UN | Y | NA | Y | Moderate |
| 2 | Canales Siguero D et al. | Y | Y | Y | Y | Y | Y | NA | Y | High |
| 3 | Canales Siguero D et al. | Y | Y | Y | Y | Y | Y | NA | Y |  |
| 4 | Jain P et al. | Y | Y | Y | UN | N | Y | NA | Y | Moderate |
| 5 | Jain P et al. | Y | Y | Y | UN | N | Y | NA | Y |  |
| 6 | Adda J et al. | Y | Y | Y | Y | N | Y | NA | Y | Moderate |
| 7 | Dina J et al. | Y | Y | Y | Y | N | Y | NA | Y | Moderate |
| 8 | Dina J et al. | Y | Y | Y | UN | N | Y | NA | Y |  |
| 9 | Dina J et al. | Y | Y | Y | UN | N | Y | NA | Y |  |
| 10 | Veronese G et al. | Y | Y | Y | Y | Y | Y | NA | Y | High |
| 11 | Hu HY et al. | Y | Y | Y | Y | N | Y | NA | Y | Moderate |
| 12 | Hu HY et al. | Y | Y | Y | Y | N | Y | NA | Y |  |
| 13 | Koehl B et al. | Y | Y | Y | Y | N | Y | NA | Y | Moderate |
| 14 | Koehl B et al. | Y | Y | Y | Y | N | Y | NA | Y |  |
| 15 | Papadogiannakis N et al. | Y | Y | Y | Y | N | Y | NA | Y | Moderate |
| 16 | Nigro G et al. | Y | Y | Y | UN | Y | Y | NA | Y | Moderate |
| 17 | Nigro G et al. | Y | Y | Y | Y | Y | Y | NA | Y |  |
| 18 | Nigro G et al. | Y | Y | Y | Y | Y | Y | NA | Y |  |
| 19 | Butin M et al. | Y | Y | Y | UN | Y | Y | NA | Y | Moderate |
| 20 | Munro K et al. | Y | Y | Y | Y | Y | Y | NA | Y | High |
| 21 | Munro K et al. | Y | Y | Y | Y | Y | Y | NA | Y |  |
| 22 | Munro K et al. | Y | Y | Y | Y | Y | Y | NA | Y |  |
| 23 | Enders G et al. | Y | Y | Y | Y | Y | Y | NA | Y | High |
| 24 | Enders G et al. | Y | Y | Y | Y | N | Y | NA | Y |  |
| 25 | Murry CE et al. | Y | Y | Y | Y | N | Y | NA | Y | Moderate |
| 26 | Dina J et al. | Y | Y | Y | Y | N | Y | NA | Y | Moderate |
| 27 | Krishnamurti L et al. | Y | Y | Y | UN | Y | Y | NA | Y | Moderate |
| 28 | Rohayem et al. | Y | Y | Y | Y | N | Y | NA | Y | Moderate |
| 29 | Zack F et al. | Y | Y | Y | Y | N | Y | NA | Y | Moderate |
| 30 | Beghetti M et al. | Y | Y | Y | Y | N | Y | NA | Y | Moderate |
| 31 | Beghetti M et al. | Y | Y | Y | UN | Y | Y | NA | Y |  |
| 32 | McMahon CJ et al. | Y | Y | Y | UN | Y | Y | NA | Y | Moderate |
| 33 | McMahon CJ et al. | Y | Y | Y | UN | Y | Y | NA | Y |  |
| 34 | O’Connell MJ et al. | Y | Y | Y | UN | N | Y | NA | UN | Low |
| 35 | Floyd A et al. | Y | Y | Y | Y | N | Y | NA | Y | Moderate |
| 36 | Ajmi H et al. | Y | Y | Y | UN | N | Y | NA | Y | Moderate |
| 37 | Spartalis M et al. | Y | Y | Y | Y | Y | Y | NA | Y | High |
| 38 | Saleh M et al. | Y | Y | Y | UN | Y | Y | NA | Y | Moderate |
| 39 | Saleh M et al. | Y | Y | Y | UN | Y | Y | NA | Y |  |
| 40 | Amabile N et al. | N | Y | N | UN | Y | Y | NA | Y | Low |
| 41 | Amabile N et al. | N | Y | N | UN | Y | Y | NA | Y |  |
| 42 | Simpson KE et al. | N | N | N | UN | UN | Y | NA | UN | Low |
| 43 | Simpson KE et al. | N | N | N | UN | UN | Y | NA | UN |  |
| 44 | Ferro V et al. | Y | N | Y | Y | N | Y | NA | Y | Moderate |
| 45 | Ferro V et al. | Y | N | Y | Y | Y | Y | NA | Y |  |
| 46 | Ferro V et al. | Y | N | Y | Y | Y | Y | NA | Y |  |
| 47 | Ferro V et al. | Y | N | Y | UN | Y | Y | NA | Y |  |
| 48 | Ferro V et al. | Y | N | Y | Y | Y | Y | NA | Y |  |
| 49 | Ferro V et al. | Y | N | Y | Y | Y | Y | NA | Y |  |
| 50 | Ferro V et al. | Y | N | Y | UN | N | Y | NA | Y |  |
| 51 | Wahjudi T et al. | Y | Y | Y | UN | UN | Y | NA | Y | Moderate |
| 52 | Wahjudi T et al. | Y | Y | Y | Y | UN | Y | NA | Y |  |
| 53 | Wahjudi T et al. | Y | Y | Y | Y | UN | Y | NA | Y |  |

* High reporting quality: ≥7 out of 8 criteria met (≥ 87.5%) → Clear, comprehensive, and well-documented cases with minimal risk of reporting bias; Moderate reporting quality: 5–6 out of 8 criteria met (62.5%–75%) → Key clinical details reported, but some methodological or diagnostic elements may be missing or unclear; Low reporting quality: <5 out of 8 criteria met (< 62.5%) → Incomplete case descriptions, often lacking essential diagnostic, therapeutic, or follow-up information.

**Abbreviations:** JBI, Joanna Briggs Institute; Y, yes; N, no; UN, unclear, NA, not applicable.
